# Crystal structure of chlorido­(2-{[2-(phenyl­car­bamo­thioyl)hydrazin-1-ylidene](pyridin-2-yl)methyl}pyridin-1-ium)gold(I) chloride sesqui­hydrate

**DOI:** 10.1107/S2056989015015480

**Published:** 2015-08-26

**Authors:** Claudia C. Gatto, Iariane J. Lima

**Affiliations:** a Laboratory of Inorganic Synthesis and Crystallography, University of Brasília, IQ, Campus Universitário Darcy Ribeiro, CEP 70904970, PO Box 4478, Brasília - DF, Brazil

**Keywords:** crystal structure, gold(I) complex, thio­semicarbazone, hydrogen bonding

## Abstract

The title complex, [AuCl(C_18_H_16_N_5_S)]Cl·1.5H_2_O, may be considered as a gold(I) compound with the corresponding metal site coordinated by a thio­semicarbazone ligand through the S atom. The ligand adopts an *E* conformation and the gold(I) atom displays the expected linear geometry with a Cl atom also bonded to the metal ion [Cl—Au—S = 174.23 (5)°]. One of the pyridyl rings is protonated, giving the gold complex an overall positive charge. Two solvent water mol­ecules, one of which is located on a twofold rotation axis, and a non-coordinating chloride ion complete the structural assembly. The mol­ecular structure is stabilized by intra­molecular and inter­molecular N—H⋯Cl, N—H⋯N, O—H⋯Cl and O—H⋯O hydrogen bonding.

## Abstract 2

Synthesis and structural characterization of a new gold(I) complex with di-2-pyridyl ketone phenyl­thio­semicarbazone, [AuCl(C_18_H_16_N_5_S)]Cl·1.5H_2_O

## Chemical context   

Thio­semicarbazones are generated from reactions of thio­semicarbazides with either an aldehyde or a ketone. They are compounds that can coordinate to transition metals and exhibit keto–enol tautomerism (Duan *et al.*, 1996 ▸). Thio­semicarbazones are known to have diverse biological activity, including anti-malarial properties and anti­bacterial, anti­tubercular, anti­viral and anti­tumor activity (Beraldo & Gambino, 2004 ▸, Casini *et al.*, 2008 ▸, Khanye *et al.*, 2010 ▸). The study of gold compounds with thio­semicarbazones has great importance: the literature reports that some compounds of this type have been shown to exhibit biological activity and have potential applications (Casini *et al.*, 2008 ▸, Lessa *et al.*, 2011 ▸).
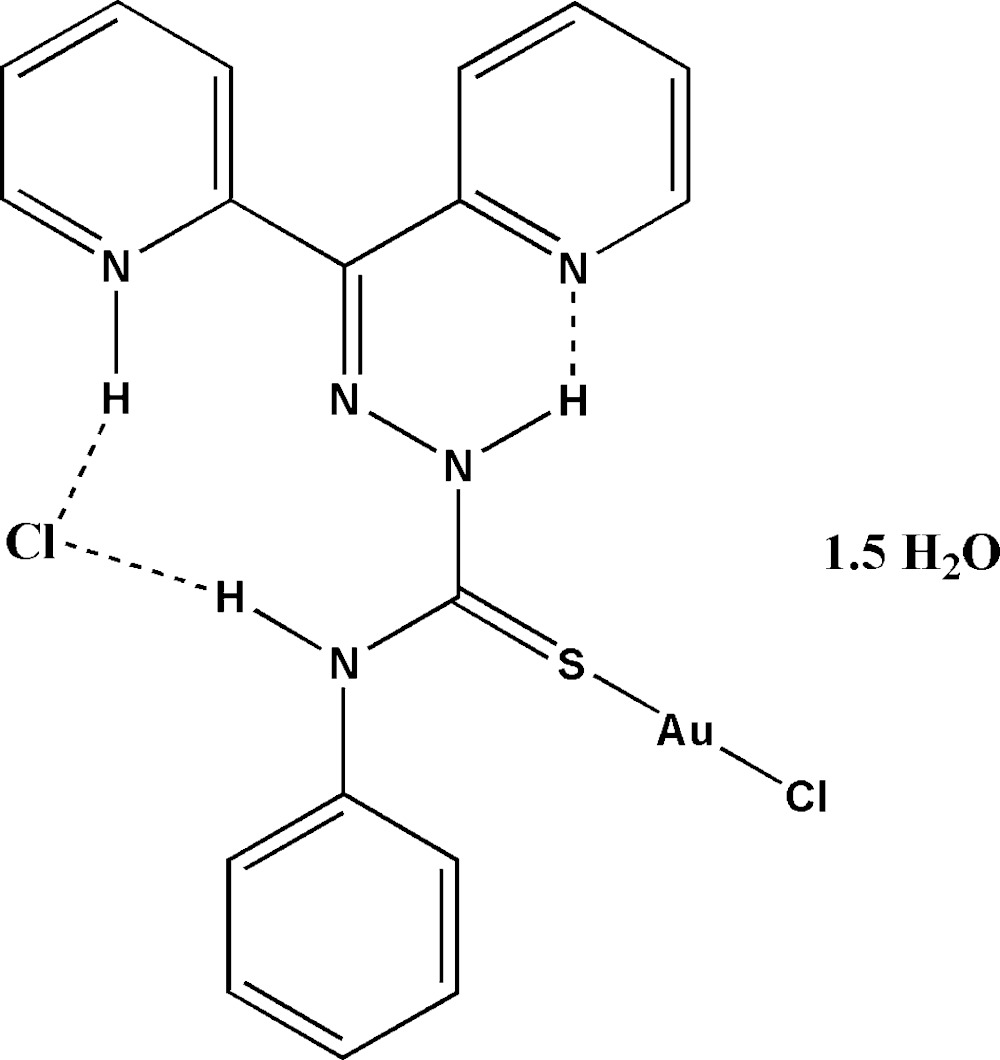


## Structural commentary   

**Figure 1:**
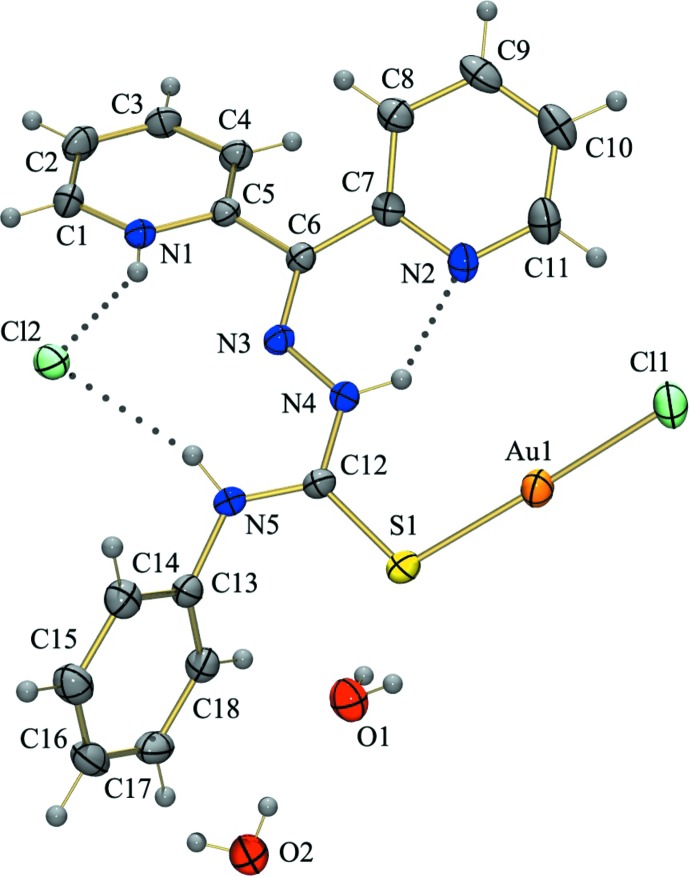
Perspective view of [AuCl(C_18_H_16_N_5_S)]Cl·1.5H_2_O with 30% probability ellipsoids and atom labeling.

In the title complex (Fig. 1 ▸), the di-2-pyridyl ketone phenyl­thio­semicarbazone ligand is protonated at the pyridine (py) nitro­gen and only the sulfur donor atom is used to bond to the central metal ion. The thio­semicarbazone adopts the *E* conformation in relation to the C6=N3 and N4—C12 bonds.

The crystal structure data confirm reduction of gold(III) of the starting material [HPy][AuCl_4_] during the synthesis. Two solvent water mol­ecules and an non-coordinating chloride ion complete the structural assembly and are hydrogen bonded to the cationic complex.

The gold(I) atom displays the expected linear geometry, with a Cl—Au—S coordination angle of 174.23 (5)°, close to the ideal angle of 180° expected for *sp* hybridization of the metal.

The C12—S1 bond length reported for di-2-pyridyl ketone phenyl­thio­semicarbazone is 1.676 (2) Å and it is lengthened to 1.713 (4) Å on coordination to gold; this is typical of the ketone form with a concomitant shortening of the N3—N4 bond (Suni *et al.*, 2006 ▸).

An intra­molecular N4—H4*A*⋯N2 hydrogen bond (Table 1 ▸) is observed.

## Supra­molecular features   

**Figure 2:**
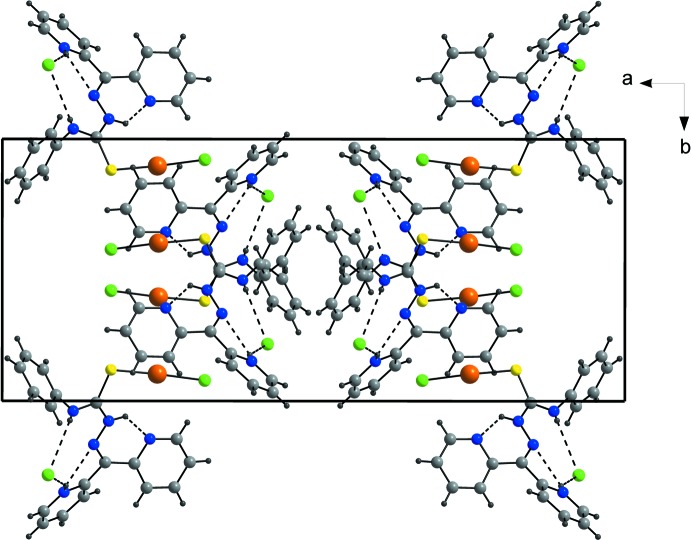
Perspective view of the compound showing the components connected by N—H⋯Cl and N—H⋯N hydrogen bonds (dashed lines), viewed along the *c* axis. Solvent water mol­ecules have been omitted for clarity.

In the crystal, the chloride ion is linked to the complex mol­ecule by N—H⋯Cl hydrogen bonds. The mol­ecular structure is also stabilized by inter­molecular O—H⋯Cl and O—H⋯O hydrogen bonding involving the water mol­ecules. Therefore, upon protonation of the ligand, hydrogen-bond formation with the chloride ion results in a stabilization of the conformation of the cationic gold complex, and hydrogen bonding plays an important role in the crystallization of the compound (Table 1 ▸ and Fig. 2 ▸).

## Related studies   

For the preparation of coordination compounds of thio­memicarbazones with gold, see: Castiñeiras *et al.* (2012 ▸); Khanye *et al.* (2010 ▸); Lessa *et al.* (2011 ▸); Sreekanth *et al.* (2004 ▸). For the spectroscopic (FT–IR) properties of thio­semi­carbazones and the crystal structure of thio­semi­carbazones, see: Beraldo & Gambino (2004 ▸); Duan *et al.* (1996 ▸); Pereiras-Gabián *et al.* (2004 ▸); Suni *et al.* (2006 ▸). For the crystal structures of di-2-pyridyl ketone phenyl­thio­semicarbazone and coordination compounds with this thio­semicarbazone, see: Bernhardt *et al.* (2009 ▸); Philip *et al.* (2005 ▸); Suni *et al.* (2006 ▸, 2007 ▸). For structure–activity studies of thio­semicarbazones, see: Bernhardt *et al.* (2009 ▸); Casini *et al.* (2008 ▸); Duan *et al.* (1996 ▸).

## Synthesis and crystallization   

Di-2-pyridyl ketone phenyl­thio­semicarbazone (1 mmol) was dissolved in about 5 ml of CH_3_CN and added to a solution of [HPy][AuCl_4_] (1 mmol) in 5 ml of CH_3_CN. A clear yellow solution was formed after heating the mixture to reflux for three h. Orange crystals deposited upon slow cooling of the solvent. Yield: 69%, m.p. 491 K. Elemental analysis, found: C, 33.71; H, 3.15; N, 10.04%; calculated for C_36_H_38_Au_2_Cl_4_N_10_O_3_S_2_: C, 33.87; H, 3.16; N, 10.97%. IR (ν_max_ cm^−1^): 3421 (O—H), 3281 (N—H), 2927 (N—H^+^), 1694 (C=N), 1150 (N—N), 765 (C=S).

## Refinement   

Crystal data, data collection and structure refinement details are summarized in Table 2 ▸. Hydrogen atoms potentially involved in hydrogen-bonding inter­actions were located in difference electron-density maps and their positional and isotropic displacement parameters were refined. Hydrogen atoms of water mol­ecules were refined with distance restraints, with an H⋯H separation of 1.38 (2) Å, the H—O distance restrained to 0.82 (2) Å and with *U*_iso_ = 1.5*U*_eq_(O). Other H atoms were included in the refinement at calculated positions and treated as riding with *U*_iso_(H) = 1.2*U*_eq_(C).

## Supplementary Material

Crystal structure: contains datablock(s) I. DOI: 10.1107/S2056989015015480/zl2637sup1.cif

Structure factors: contains datablock(s) I. DOI: 10.1107/S2056989015015480/zl2637Isup3.hkl

CCDC reference: 1419509

Additional supporting information:  crystallographic information; 3D view; checkCIF report

## Figures and Tables

**Table 1:** Hydrogen-bond geometry (, )

*D*H*A*	*D*H	H*A*	*D* *A*	*D*H*A*
N5H5*A*Cl2	0.86	2.46	3.246(4)	153
N4H4*A*N2	0.86	1.97	2.629(5)	133
N1H1*A*Cl2	0.80(4)	2.26(4)	2.989(4)	150(4)
O1H1*W*1Cl1^i^	0.80(2)	2.70(5)	3.353(4)	140(6)
O1H1*W*2Cl2^ii^	0.81(2)	2.39(2)	3.206(4)	177(6)
O2H2*W*2O1	0.82(2)	2.06(3)	2.855(5)	163(7)

Symmetry codes: (i) 
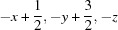
; (ii) 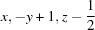.

**Table 2:** Experimental details

Crystal data	
Chemical formula	[AuCl(C_18_H_16_N_5_S)]Cl1.5H_2_O
*M*_r_	629.31
Crystal system, space group	Monoclinic, *C*2/*c*
Temperature (K)	296
*a*, *b*, *c* ()	31.0939(7), 12.2704(3), 11.8851(3)
()	110.174(1)
*V* (^3^)	4256.38(18)
*Z*	8
Radiation type	Mo *K*
(mm^1^)	7.28
Crystal size (mm)	0.24 0.22 0.14

Data collection	
Diffractometer	Bruker CCD SMART APEXII
Absorption correction	Multi-scan (*SADABS*; Bruker, 2009 ▸)
*T*_min_, *T*_max_	0.274, 0.429
No. of measured, independent and observed [*I* > 2(*I*)] reflections	15424, 4345, 3220
*R*_int_	0.038
(sin /)_max_ (^1^)	0.626

Refinement	
*R*[*F* ^2^ > 2(*F* ^2^)], *wR*(*F* ^2^), *S*	0.031, 0.072, 0.97
No. of reflections	4345
No. of parameters	271
No. of restraints	3
H-atom treatment	H atoms treated by a mixture of independent and constrained refinement
_max_, _min_ (e ^3^)	0.97, 0.78

Computer programs: *APEX2* and *SAINT* (Bruker, 2009 ▸), *SHELXTL* (Sheldrick, 2008 ▸), *SHELXL2014*/7 (Sheldrick, 2015 ▸ ▸), *DIAMOND* (Crystal Impact, 2014 ▸) and *publCIF* (Westrip, 2010 ▸).

## supplementary crystallographic information

### Crystal data

**Table:**

[AuCl(C_18_H_16_N_5_S)]Cl·1.5H_2_O	*D*_x_ = 1.964 Mg m^−^^3^
*M**_r_* = 629.31	Melting point: 491 K
Monoclinic, *C*2/*c*	Mo *K*α radiation, λ = 0.71073 Å
*a* = 31.0939 (7) Å	Cell parameters from 4811 reflections
*b* = 12.2704 (3) Å	θ = 2.7–25.2°
*c* = 11.8851 (3) Å	µ = 7.28 mm^−^^1^
β = 110.174 (1)°	*T* = 296 K
*V* = 4256.38 (18) Å^3^	Block, red
*Z* = 8	0.24 × 0.22 × 0.14 mm
*F*(000) = 2424	

### Data collection

**Table:**

Bruker CCD SMART APEXII diffractometer	3220 reflections with *I* > 2σ(*I*)
Radiation source: fine-focus sealed tube	*R*_int_ = 0.038
phi & ω scans	θ_max_ = 26.4°, θ_min_ = 1.8°
Absorption correction: multi-scan (*SADABS*; Bruker, 2009)	*h* = −37→38
*T*_min_ = 0.274, *T*_max_ = 0.429	*k* = −14→15
15424 measured reflections	*l* = −14→14
4345 independent reflections	

### Refinement

**Table:**

Refinement on *F*^2^	Primary atom site location: structure-invariant direct methods
Least-squares matrix: full	Secondary atom site location: difference Fourier map
*R*[*F*^2^ > 2σ(*F*^2^)] = 0.031	Hydrogen site location: mixed
*wR*(*F*^2^) = 0.072	H atoms treated by a mixture of independent and constrained refinement
*S* = 0.97	*w* = 1/[σ^2^(*F*_o_^2^) + (0.0364*P*)^2^] where *P* = (*F*_o_^2^ + 2*F*_c_^2^)/3
4345 reflections	(Δ/σ)_max_ = 0.001
271 parameters	Δρ_max_ = 0.97 e Å^−^^3^
3 restraints	Δρ_min_ = −0.78 e Å^−^^3^

### Special details

**Table:**

Geometry. All e.s.d.'s (except the e.s.d. in the dihedral angle between two l.s. planes) are estimated using the full covariance matrix. The cell e.s.d.'s are taken into account individually in the estimation of e.s.d.'s in distances, angles and torsion angles; correlations between e.s.d.'s in cell parameters are only used when they are defined by crystal symmetry. An approximate (isotropic) treatment of cell e.s.d.'s is used for estimating e.s.d.'s involving l.s. planes.

### Fractional atomic coordinates and isotropic or equivalent isotropic displacement parameters (Å^2^)

**Table:**

	*x*	*y*	*z*	*U*_iso_*/*U*_eq_	
Au1	0.25092 (2)	0.60284 (2)	0.04569 (2)	0.04892 (9)	
S1	0.32514 (5)	0.61867 (10)	0.16165 (13)	0.0581 (4)	
Cl1	0.17486 (5)	0.57863 (11)	−0.05533 (12)	0.0610 (4)	
C12	0.34886 (15)	0.4928 (3)	0.1607 (4)	0.0388 (10)	
N3	0.35315 (13)	0.3345 (3)	0.0578 (3)	0.0370 (9)	
N5	0.38617 (12)	0.4607 (3)	0.2458 (3)	0.0424 (9)	
H5A	0.3953	0.3954	0.2403	0.051*	
N4	0.32925 (13)	0.4234 (3)	0.0690 (3)	0.0401 (9)	
H4A	0.3022	0.4355	0.0190	0.048*	
C13	0.41343 (15)	0.5235 (4)	0.3473 (4)	0.0410 (11)	
C5	0.36773 (15)	0.1810 (3)	−0.0344 (4)	0.0355 (10)	
C6	0.33459 (15)	0.2653 (3)	−0.0268 (3)	0.0342 (10)	
C7	0.28703 (16)	0.2612 (3)	−0.1109 (3)	0.0376 (10)	
C4	0.36887 (17)	0.1356 (3)	−0.1392 (4)	0.0431 (11)	
H4	0.3469	0.1549	−0.2121	0.052*	
C1	0.43375 (18)	0.0813 (3)	0.0737 (5)	0.0479 (12)	
H1	0.4560	0.0642	0.1470	0.057*	
C18	0.43406 (17)	0.6200 (4)	0.3336 (5)	0.0472 (12)	
H18	0.4292	0.6490	0.2579	0.057*	
C15	0.45009 (18)	0.5339 (4)	0.5600 (4)	0.0560 (14)	
H15	0.4557	0.5049	0.6360	0.067*	
C16	0.47070 (18)	0.6300 (4)	0.5471 (5)	0.0585 (14)	
H16	0.4903	0.6657	0.6142	0.070*	
C14	0.42124 (16)	0.4803 (4)	0.4608 (4)	0.0481 (12)	
H14	0.4071	0.4158	0.4697	0.058*	
C17	0.46232 (17)	0.6725 (4)	0.4362 (5)	0.0553 (13)	
H17	0.4758	0.7384	0.4286	0.066*	
C3	0.40278 (18)	0.0613 (4)	−0.1356 (4)	0.0492 (12)	
H3	0.4037	0.0304	−0.2062	0.059*	
C2	0.43484 (18)	0.0335 (4)	−0.0286 (5)	0.0536 (13)	
H2	0.4573	−0.0177	−0.0254	0.064*	
N2	0.26250 (14)	0.3552 (3)	−0.1220 (3)	0.0471 (10)	
N1	0.40084 (13)	0.1526 (3)	0.0690 (3)	0.0393 (9)	
C9	0.22292 (19)	0.1715 (5)	−0.2508 (5)	0.0588 (14)	
H9	0.2095	0.1095	−0.2936	0.071*	
C8	0.26759 (17)	0.1682 (4)	−0.1724 (4)	0.0486 (12)	
H8	0.2844	0.1038	−0.1612	0.058*	
C11	0.21949 (19)	0.3548 (5)	−0.1966 (4)	0.0586 (14)	
H11	0.2024	0.4181	−0.2030	0.070*	
C10	0.19855 (19)	0.2663 (5)	−0.2653 (4)	0.0592 (14)	
H10	0.1688	0.2710	−0.3197	0.071*	
Cl2	0.42609 (4)	0.21756 (9)	0.32609 (9)	0.0506 (3)	
O1	0.42303 (14)	0.7870 (4)	0.0930 (3)	0.0663 (10)	
H1W1	0.3956 (7)	0.787 (6)	0.069 (6)	0.099*	
H1W2	0.424 (2)	0.783 (5)	0.025 (3)	0.099*	
O2	0.5000	0.9028 (5)	0.2500	0.087 (2)	
H2W2	0.4803 (19)	0.859 (4)	0.213 (6)	0.130*	
H1A	0.3997 (14)	0.186 (3)	0.126 (4)	0.033 (13)*	

### Atomic displacement parameters (Å^2^)

**Table:**

	*U*^11^	*U*^22^	*U*^33^	*U*^12^	*U*^13^	*U*^23^
Au1	0.04494 (14)	0.05060 (13)	0.04726 (13)	0.01206 (9)	0.01084 (10)	−0.00251 (9)
S1	0.0458 (8)	0.0439 (7)	0.0735 (9)	0.0109 (6)	0.0064 (7)	−0.0198 (6)
Cl1	0.0451 (8)	0.0752 (9)	0.0550 (8)	0.0134 (6)	0.0075 (7)	0.0022 (6)
C12	0.042 (3)	0.032 (2)	0.043 (2)	0.001 (2)	0.014 (2)	−0.005 (2)
N3	0.041 (2)	0.033 (2)	0.0358 (19)	0.0044 (16)	0.0122 (18)	−0.0029 (16)
N5	0.043 (3)	0.0311 (19)	0.047 (2)	0.0033 (16)	0.008 (2)	−0.0065 (17)
N4	0.033 (2)	0.037 (2)	0.046 (2)	0.0049 (16)	0.0089 (19)	−0.0062 (17)
C13	0.036 (3)	0.038 (2)	0.047 (3)	0.004 (2)	0.012 (2)	−0.004 (2)
C5	0.040 (3)	0.030 (2)	0.036 (2)	−0.0010 (19)	0.012 (2)	0.0018 (19)
C6	0.038 (3)	0.033 (2)	0.033 (2)	0.0017 (18)	0.013 (2)	0.0008 (19)
C7	0.041 (3)	0.042 (2)	0.032 (2)	0.000 (2)	0.016 (2)	0.002 (2)
C4	0.052 (3)	0.040 (2)	0.037 (2)	0.005 (2)	0.015 (2)	−0.002 (2)
C1	0.050 (3)	0.040 (3)	0.047 (3)	0.008 (2)	0.007 (3)	0.003 (2)
C18	0.046 (3)	0.043 (3)	0.050 (3)	0.004 (2)	0.014 (3)	0.003 (2)
C15	0.056 (4)	0.066 (3)	0.044 (3)	0.002 (3)	0.015 (3)	−0.008 (3)
C16	0.040 (3)	0.066 (3)	0.060 (3)	0.000 (3)	0.006 (3)	−0.023 (3)
C14	0.045 (3)	0.050 (3)	0.052 (3)	0.000 (2)	0.019 (3)	0.001 (2)
C17	0.046 (3)	0.041 (3)	0.075 (4)	−0.005 (2)	0.016 (3)	−0.012 (3)
C3	0.056 (3)	0.043 (3)	0.050 (3)	0.003 (2)	0.020 (3)	−0.012 (2)
C2	0.053 (3)	0.044 (3)	0.069 (4)	0.011 (2)	0.027 (3)	−0.003 (3)
N2	0.043 (3)	0.054 (2)	0.040 (2)	0.0091 (19)	0.008 (2)	0.0055 (18)
N1	0.044 (3)	0.033 (2)	0.037 (2)	0.0027 (17)	0.010 (2)	−0.0038 (18)
C9	0.045 (3)	0.073 (4)	0.054 (3)	−0.024 (3)	0.012 (3)	−0.006 (3)
C8	0.045 (3)	0.050 (3)	0.051 (3)	−0.006 (2)	0.016 (3)	−0.001 (2)
C11	0.048 (4)	0.076 (4)	0.047 (3)	0.013 (3)	0.009 (3)	0.008 (3)
C10	0.041 (3)	0.088 (4)	0.044 (3)	−0.006 (3)	0.009 (3)	−0.001 (3)
Cl2	0.0610 (9)	0.0450 (6)	0.0386 (6)	−0.0022 (6)	0.0080 (6)	0.0000 (5)
O1	0.065 (3)	0.079 (2)	0.051 (2)	0.002 (2)	0.015 (2)	0.003 (2)
O2	0.077 (5)	0.064 (4)	0.092 (5)	0.000	−0.004 (4)	0.000

### Geometric parameters (Å, º)

**Table:**

Au1—S1	2.2515 (14)	C18—H18	0.9300
Au1—Cl1	2.2725 (14)	C15—C16	1.376 (7)
S1—C12	1.713 (4)	C15—C14	1.377 (6)
C12—N5	1.309 (5)	C15—H15	0.9300
C12—N4	1.351 (5)	C16—C17	1.357 (7)
N3—C6	1.290 (5)	C16—H16	0.9300
N3—N4	1.353 (5)	C14—H14	0.9300
N5—C13	1.436 (5)	C17—H17	0.9300
N5—H5A	0.8600	C3—C2	1.362 (7)
N4—H4A	0.8600	C3—H3	0.9300
C13—C18	1.383 (6)	C2—H2	0.9300
C13—C14	1.391 (6)	N2—C11	1.325 (6)
C5—N1	1.349 (5)	N1—H1A	0.80 (4)
C5—C4	1.375 (6)	C9—C10	1.366 (7)
C5—C6	1.485 (6)	C9—C8	1.381 (7)
C6—C7	1.473 (6)	C9—H9	0.9300
C7—N2	1.364 (5)	C8—H8	0.9300
C7—C8	1.378 (6)	C11—C10	1.380 (7)
C4—C3	1.384 (6)	C11—H11	0.9300
C4—H4	0.9300	C10—H10	0.9300
C1—N1	1.332 (6)	O1—H1W1	0.80 (2)
C1—C2	1.361 (7)	O1—H1W2	0.813 (19)
C1—H1	0.9300	O2—H2W2	0.82 (2)
C18—C17	1.390 (6)		
			
S1—Au1—Cl1	174.23 (5)	C16—C15—H15	119.9
C12—S1—Au1	105.72 (16)	C14—C15—H15	119.9
N5—C12—N4	117.8 (4)	C17—C16—C15	119.8 (5)
N5—C12—S1	122.3 (3)	C17—C16—H16	120.1
N4—C12—S1	119.8 (3)	C15—C16—H16	120.1
C6—N3—N4	119.6 (4)	C15—C14—C13	119.5 (5)
C12—N5—C13	126.6 (4)	C15—C14—H14	120.2
C12—N5—H5A	116.7	C13—C14—H14	120.2
C13—N5—H5A	116.7	C16—C17—C18	121.7 (5)
C12—N4—N3	118.5 (4)	C16—C17—H17	119.2
C12—N4—H4A	120.7	C18—C17—H17	119.2
N3—N4—H4A	120.7	C2—C3—C4	119.9 (5)
C18—C13—C14	120.5 (4)	C2—C3—H3	120.1
C18—C13—N5	121.6 (4)	C4—C3—H3	120.1
C14—C13—N5	117.7 (4)	C1—C2—C3	119.4 (5)
N1—C5—C4	118.1 (4)	C1—C2—H2	120.3
N1—C5—C6	116.9 (4)	C3—C2—H2	120.3
C4—C5—C6	124.9 (4)	C11—N2—C7	117.6 (4)
N3—C6—C7	128.7 (4)	C1—N1—C5	122.8 (4)
N3—C6—C5	111.9 (4)	C1—N1—H1A	124 (3)
C7—C6—C5	119.4 (4)	C5—N1—H1A	113 (3)
N2—C7—C8	121.4 (4)	C10—C9—C8	119.7 (5)
N2—C7—C6	115.7 (4)	C10—C9—H9	120.2
C8—C7—C6	122.8 (4)	C8—C9—H9	120.2
C5—C4—C3	119.7 (4)	C7—C8—C9	119.2 (5)
C5—C4—H4	120.1	C7—C8—H8	120.4
C3—C4—H4	120.1	C9—C8—H8	120.4
N1—C1—C2	120.0 (5)	N2—C11—C10	124.1 (5)
N1—C1—H1	120.0	N2—C11—H11	118.0
C2—C1—H1	120.0	C10—C11—H11	118.0
C13—C18—C17	118.2 (5)	C9—C10—C11	117.9 (5)
C13—C18—H18	120.9	C9—C10—H10	121.1
C17—C18—H18	120.9	C11—C10—H10	121.1
C16—C15—C14	120.3 (5)	H1W1—O1—H1W2	92 (6)
			
Au1—S1—C12—N5	−157.3 (4)	N5—C13—C18—C17	−175.9 (4)
Au1—S1—C12—N4	23.6 (4)	C14—C15—C16—C17	0.4 (8)
N4—C12—N5—C13	176.7 (4)	C16—C15—C14—C13	0.7 (8)
S1—C12—N5—C13	−2.5 (7)	C18—C13—C14—C15	−0.6 (7)
N5—C12—N4—N3	−12.1 (6)	N5—C13—C14—C15	175.0 (4)
S1—C12—N4—N3	167.0 (3)	C15—C16—C17—C18	−1.6 (8)
C6—N3—N4—C12	177.8 (4)	C13—C18—C17—C16	1.6 (8)
C12—N5—C13—C18	−60.4 (7)	C5—C4—C3—C2	−0.1 (8)
C12—N5—C13—C14	124.1 (5)	N1—C1—C2—C3	1.7 (8)
N4—N3—C6—C7	−7.3 (6)	C4—C3—C2—C1	−1.5 (8)
N4—N3—C6—C5	173.0 (3)	C8—C7—N2—C11	−1.7 (6)
N1—C5—C6—N3	31.7 (5)	C6—C7—N2—C11	−179.8 (4)
C4—C5—C6—N3	−143.8 (4)	C2—C1—N1—C5	−0.2 (7)
N1—C5—C6—C7	−148.1 (4)	C4—C5—N1—C1	−1.4 (7)
C4—C5—C6—C7	36.4 (6)	C6—C5—N1—C1	−177.2 (4)
N3—C6—C7—N2	18.1 (6)	N2—C7—C8—C9	2.8 (7)
C5—C6—C7—N2	−162.1 (4)	C6—C7—C8—C9	−179.2 (4)
N3—C6—C7—C8	−160.0 (4)	C10—C9—C8—C7	−0.7 (7)
C5—C6—C7—C8	19.7 (6)	C7—N2—C11—C10	−1.6 (7)
N1—C5—C4—C3	1.5 (7)	C8—C9—C10—C11	−2.3 (8)
C6—C5—C4—C3	176.9 (4)	N2—C11—C10—C9	3.6 (8)
C14—C13—C18—C17	−0.5 (7)		

### Hydrogen-bond geometry (Å, º)

**Table:**

*D*—H···*A*	*D*—H	H···*A*	*D*···*A*	*D*—H···*A*
N5—H5*A*···Cl2	0.86	2.46	3.246 (4)	153
N4—H4*A*···N2	0.86	1.97	2.629 (5)	133
N1—H1*A*···Cl2	0.80 (4)	2.26 (4)	2.989 (4)	150 (4)
O1—H1*W*1···Cl1^i^	0.80 (2)	2.70 (5)	3.353 (4)	140 (6)
O1—H1*W*2···Cl2^ii^	0.81 (2)	2.39 (2)	3.206 (4)	177 (6)
O2—H2*W*2···O1	0.82 (2)	2.06 (3)	2.855 (5)	163 (7)

Symmetry codes: (i) −*x*+1/2, −*y*+3/2, −*z*; (ii) *x*, −*y*+1, *z*−1/2.
